# Detection of *Avibacterium paragallinarum* by Polymerase chain reaction from outbreaks of Infectious coryza of poultry in Andhra Pradesh

**DOI:** 10.14202/vetworld.2015.103-108

**Published:** 2015-01-29

**Authors:** T. M. Nabeel Muhammad, B. Sreedevi

**Affiliations:** Department of Veterinary Epidemiology and Preventive Medicine, College of Veterinary Science, Sri Venkateswara Veterinary University, Tirupati, Andhra Pradesh, India

**Keywords:** infectious coryza, polymerase chain reaction, poultry

## Abstract

**Aim:**

This study was carried out for the detection of *Avibacterium paragallinarum* from outbreaks of infectious coryza of poultry

**Materials and Methods:**

The polymerase chain reaction (PCR) was standardized for the diagnosis of infectious coryza by using infectious coryza Killed vaccine, ventri biologicals, Pune as source of DNA of *A. paragallinarum*. Five outbreaks of infectious coryza from Andhra Pradesh were investigated in the present study. A total of 56 infra orbital sinus swabs and 22 nasal swabs were tested by PCR.

**Results:**

PCR analysis showed 56 positives (71.7%) for infectious coryza out of total 78 samples tested. Of 56 infra orbital sinus swabs tested, 47 were positive (83.9%) and 9 nasal swabs (40.9%) out of 22 tested had given positive results for infectious coryza. Samples collected from birds at acute stage of disease and samples collected before treatment with antibiotics were given better results on PCR.

**Conclusion:**

For preventing the economic losses associated with the disease, an early, accurate and rapid diagnosis is essential. PCR is a rapid and highly sensitive diagnostic technique which can substitute conventional cultural examination.

## Introduction

A group of respiratory diseases, often called as respiratory disease complex which produces closely resembling symptoms, mixed infections of respiratory system with multiple etiologies are contributing to the complexity in the proper diagnosis and differentiation of respiratory diseases. Infectious coryza is a respiratory disease of chickens caused by the bacterium, *Avibacterium paragallinarum* primarily affecting upper respiratory tract, including the involvement of nasal passages, infra orbital and paranasal sinuses.

Infectious coryza is a cosmopolitan disease, which has been reported from all around the world where chickens are raised including India. The economic losses associated with infectious coryza results from poor growth performance in growing birds including broilers, marked reduction (10-40%) in egg production in layers and increased culling rates in meat chickens. Chronically infected birds or recovered healthy birds act as reservoirs of infection in a population and makes the disease endemic in an area [1,2].

The disease is recognized as a cause of significant loss to the poultry industry all over the world. For reducing the economic losses associated with this disease, early, rapid and accurate diagnosis is essential. In developing countries, conventional diagnosis of infectious coryza is based on clinical signs, demonstration of satellite colonies by cultural examination and confirmation is by biochemical tests. However, the factors like simultaneous occurrence of combined respiratory infections, occurrence of NAD independent strains, overgrowth of fast growing bacteria, which are masking the growth of *A. paragallinarum*, requirement of special media for culturing, presence of different biovars, etc. makes the confirmatory diagnosis difficult. Hence, nucleic acid based techniques are the best alternative tools in the easy and rapid confirmatory diagnosis.

The present study was taken up to detect *A. paragallinarum* by Polymerase chain reaction from outbreaks of Infectious coryza of poultry in Andhra Pradesh

## Materials and Methods

### Ethical approval

All samples were collected as per standard collection procedure.

### Collection of samples

The samples used in the study were collected from suspected outbreaks of infectious coryza from Guntur, Krishna, West Godavari and Chittoor districts of Andhra Pradesh according to the method described previously [3] in which 56 samples were infra orbital sinus swabs and 22 were nasal swabs. Samples consisted of swabs from three commercial poultry farms and two backyard flocks of Aseel chicken of different age groups with no history of vaccination against infectious coryza. Swabs were collected aseptically and soaked in 30% glycerol-phosphate buffered saline [4]. The samples were transported to the laboratory in ice pack at the earliest and stored at −20°C.

### DNA extraction

The standard phenol- chloroform method described previously [5] was employed for the extraction of *A. paragallinarum* DNA with necessary modifications. Briefly, 567 μl of sample was mixed with 30 μl of 10% Sodium Do-decyl Sulfate (SDS) and 3 μl of proteinase-K (20 μg/ml) and incubated at 37°C for one hour. Equal volume of phenol: Chloroform solution was added and vortexed properly and centrifuged at 13000 rpm for 10 minutes at 4°C. The supernatant was taken out carefully and the phenol-chloroform extraction was repeated. Final supernatant was taken and mixed with 2.5 volume of chilled absolute ethanol and 1/10th volume of 3M Sodium acetate (pH-5.2) and kept at −20°C for overnight. The tube was centrifuged at 13000 rpm for 10 min, the pellet was washed with 70% chilled ethanol, air dried and dissolved in 30 μl of tris-ethylenediaminetetraacetic acid (TE) buffer and stored at −20°C until use.

The purity of the extracted DNA was assessed by spectrophotometer. The DNA was diluted in TE buffer and absorbency at 260 nm and 280 nm was recorded. The ratio of the absorbance at 260 and 280 nm was calculated. The sample giving a ratio of 1.8 or above was considered as pure DNA and used for further steps of the study [5]. Quantification of DNA was carried out by using Ethidium bromide binding assay and U.V. spectrophotometer reading as per the procedure out lined previously [5]. The DNA was diluted to 5 ng per μl of TE buffer. The DNA extracted from the infectious coryza killed vaccine (Ventri Biologicals, Pune) was used as standard DNA in the present study. It was used for standardization of polymerase chain reaction (PCR) protocol and as positive control in PCR test for field samples.

### PCR

The primers described by previous works were used in the present study [6]. The sequence was TGA GGG TAG TCT TGC ACG CGA AT (23 bp) for forward primer and CAA GGT ATC GAT CGT CTC TCT ACT (24 bp) for the reverse primer. The Red Dye PCR Master mix (Genei, Bangalore) was used for PCR reaction which contains premixed dNTPs, Taq polymerase, MgCl_2_ and buffer at optimum concentrations. The gel loading dye was also incorporated to the master mix. About 25 μl reactions were used and the protocol was initially standardized for optimizing the concentration of components of the reaction mixture in the PCR assay and then by varying the annealing temperature and cycling conditions [6] using Kyratec Supercycler SC200 thermocycler. The PCR product was stored at -20°C until use.

The standardized protocol was used in PCR for field samples collected. The reaction volume used was 25 μl in which 12.5 μl of red dye master mix along with 3 μl of target DNA, 0.5 μl each of primers and 8.5 μl of molecular biology grade water were used. Initially the tubes were exposed to 94°C for 2.5 min for denaturation. Then 30 cycles of denaturation at 94°C (1 min), annealing at 58°C (1 min), extension at 72°C (2 min) was carried out. The reaction was at 72°C for 10 min for final elongation before bringing to the final holding temperature of 4°C.

The PCR amplified product was analyzed by electrophoresis in two percent agarose gels and visualized by ethidium bromide staining. About 10 μl of the PCR product along with gel loading dye was loaded to the wells. The electrophoresis was performed at a voltage of 5 Volt/cm of the gel. After sufficient migration, the gels were taken to gel documentation system (Alpha Innotech) and the results were recorded. Appropriate positive and negative controls were included in the PCR reaction.

## Results

**Figure-1:**
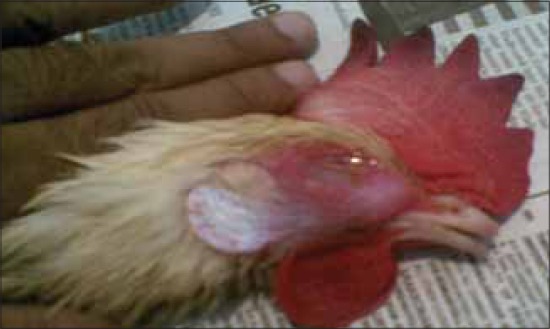
A layer bird showing ocular discharge and infra orbital sinus swelling

**Figure-2:**
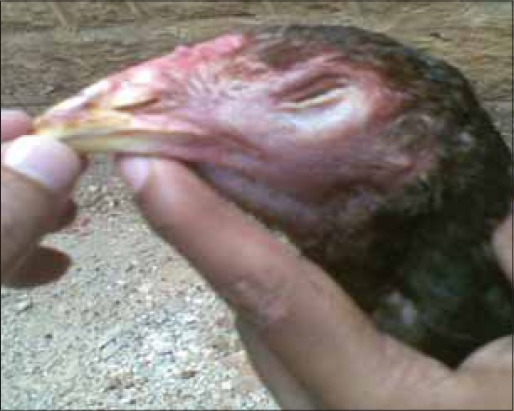
An adult Aseel bird suffering from infectious coryza showing facial oedema and sinus swelling

**Figure-3:**
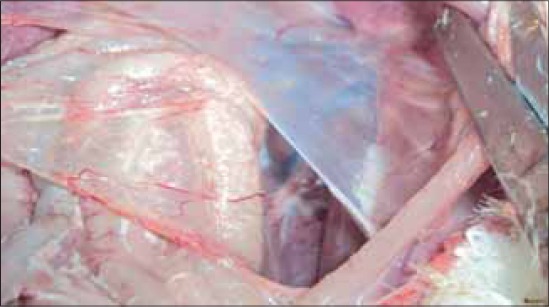
Airsacculitis in a commercial chicken suffering from infectious coryza

During the present study, five suspected outbreaks of infectious coryza from Andhra Pradesh were investigated which included outbreaks in commercial poultry and native Aseel chicken. The birds were showing signs of acute upper respiratory tract infections like coughing, sneezing, nasal discharge, facial edema, edema of wattle and comb and lacrimation and conjunctivitis. Anorexia and prominent infra orbital sinus swelling were observed. Most prominent features of infectious coryza are an acute inflammation of the upper respiratory tract including the involvement of nasal passages and sinus with a serous to mucoid nasal discharge, facial edema and conjunctivitis [Figures-1-3].

Outbreaks were reported from two layer farms which were following multi-age farming with a morbidity of 25-30% and the mortality of 5% to 10%. Morbidity of 40% and mortality of 10% were recorded along with reduced growth rate in the broiler farm from which was a small scale farm with a flock size of 20, 000. Poor hygienic and biosecurity measures were observed and this farm had the history of infectious coryza outbreak in the previous batch also. Generally, infectious coryza is characterized by high morbidity and low mortality with a drop of 10-40% in egg production. The higher production losses could be because of the stress on the birds, climatic conditions, presence of opportunistic pathogens, bio security and hygiene of the farm, parasitism etc. The Chronic or healthy carrier birds were recognized as the main reservoir of the infection and the multi-aged farms are at a higher risk of infectious coryza because of this reason. During the post-mortem examination, there was no characteristic feature of any other respiratory viral pathogen. Infra orbital sinus swelling typical to infectious coryza was very evident. The commercial flocks were vaccinated against Newcastle disease (ND), Infectious Bronchitis, Infectious Bursal Disease. The Aseel flocks were vaccinated against ND. No attempt was made to isolate any viral pathogens.

### DNA extraction

The presence of DNA extracted from vaccine was confirmed by agarose gel electrophoresis followed by ethidium bromide staining. When viewed under UV transilluminator, a single band of DNA was observed in the gel, just below the well in all the samples. The A260/A280 ratio of 1.8 or more was obtained for all the samples from vaccine.

### Standardization of PCR

PCR was carried out as described under materials and methods. The size of the amplified product was analyzed by agarose gel electrophoresis using standard DNA molecular size marker. The size of the amplified product was 500 bp, which was the size of the amplicon defined by selected primers. No amplification was observed in negative control indicating that amplicon was specific to bacteria *A. paragallinarum*.

### Screening of field samples for infectious coryza by applying PCR

**Figure-4:**
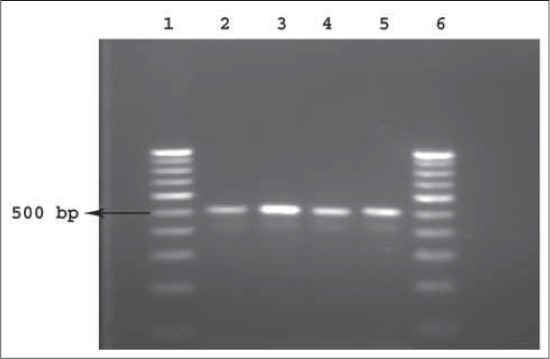
500 bp polymerase chain reaction (PCR) amplification product of *Avibacterium paragellinarum* from infectious coryza field samples. Line 1 and 6: 100 bp molecular weight marker, Lane 2-5: PCR amplified product of *A. paragellinarum*

The standardized protocol was used to screen the field samples. The results of PCR analysis showed 56 positives (71.7%) for infectious coryza out of total 78 samples tested. Out of 56 infra orbital sinus swabs tested, 47 were positive (83.9%) and 9 nasal swabs (40.9%) out of 22 tested had given positive results for infectious coryza [Figure-4]. Samples from Vijayawada outbreak gave 100% (15 out of 15) positivity for infra orbital sinus and 57% (four out of seven) positives for nasal swabs. Samples from Tenali showed 93.3% (14 out of 15) positivity for infra orbital sinus and 60% (three out of five) positivity for nasal swabs. Outbreak from Tirupati showed 83.3% (10 out of 12) positivity for infra orbital sinus swabs and all the nasal swabs were negative. Samples collected from BN Kandriga showed 80% (eight out of 10) positivity for infra orbital sinus swabs and 50% positivity for nasal swabs. All the samples tested from Dwarapudi gave negative results in PCR [Table-1]. The combined PCR results of both infra orbital sinus swabs and nasal swabs showed 86.3% positive samples from Vijayawada (19 out of 22) followed by 85% from Tenali (17 out of 20), 71.4% each from Tirupati and BN Kandriga (10 out of 14 each). Out of the total 74 infra orbital sinus swabs tested, PCR gave 47 (83.9%) positive results whereas cultural examination showed only 20 (40%) positive results (details of cultural examination of samples not mentioned in this article).

**Table-1:** The results of screening of infectious coryza suspected field samples by PCR

Outbreak No.	Place	District	No. of samples Screened by PCR			No. of samples positive in PCR		
	
			Infra orbital sinus swabs	Nasal swabs	Total	Infra orbital sinus swabs (%)	Nasal swabs (%)	Total
1	Vijayawada	Krishna	15	7	22	15 (100)	4 (57)	19 (86.3)
2	Dwarapudi	West Godavari	4	4	8	0 (0)	0 (0)	0 (0)
3	Tenali	Guntur	15	5	20	14 (93.3)	3 (60)	17 (85)
4	Tirupati	Chittoor	12	2	14	10 (83.3)	0 (0)	10 (71.4)
5	BN Kandriga	Chittoor	10	4	14	8 (80)	2 (50)	10 (71.4)
Total			56	22	78	47 (83.9)	9 (40.9)	56 (71.7)

PCR: Polymerase chain reaction

## Discussion

Infectious coryza is an upper respiratory tract disease of poultry with considerable economic impact, particularly in multi aged farms. The isolation and identification of the causative agent, of *A. paragallinarum* is difficult and demanding. The conventional diagnosis of infectious coryza is based on the appearance of typical clinical signs, isolation of satellitic organisms and further biochemical characterization [3,7]. But the dependence or the hemophilic nature of (requirement of V factor or NAD) *A. paragallinarum* is complicated by many facts. *A. paragallinarum* is a slow growing organism which will take 36-48 h or even more time to show detectable colonies. But the vigorous growth of the bacteria, which are in co-infection will mask the growth of the *A. paragallinarum* and the satellitic growth may not be appreciated. Two nonpathogenic, hemophilus bacteria named *Avibacterium avium* and *Avibacterium volatinum*, which are the part of normal flora of the chicken show satellitic colony growth similar to that of *A. paragallinarum* on blood agar. The reports of emergence of NAD-independent *A. paragallinarum*, which will not show satellitic growth again complicated this conventional method of identification [6].

Diagnosis of the infectious coryza can be more complicated when it co-occurs with other pathogens, especially bacteria like *Pasteurella multocida, Ornithobacterium rhinotracheale, Salmonella* species etc. [8,9]. Further complications can be contributed by the presence of opportunistic pathogens like *Escherichia coli, Pseudomonas, Proteus*, *Staphylococcus* species, *Streptococcus* species, *Corynebacterium* etc. during the cultural examination of samples from suspected infectious coryza cases [10-13]. Typical isolates of the *A. paragallinarum* have strict nutritional demands when grown *in-vitro*, meaning that complex media with costly ingredients such as NAD, oleic albumin complex, chicken serum and thiamine must be used to obtain pure cultures [3]. Some complex media, like supplemented test medium agar (TM/SN) and Hemophilus maintenance medium described previously are proven useful for characterization tests following isolation but not suitable for isolation [14].

The difficulties associated with conventional culture method and biochemical characterization of infectious coryza made the molecular technique, PCR attractive. There was no standard culture of *A. paragallinarum* readily available in the country and obtaining it from other countries was very difficult due to strict biosecurity norms adopted in the country. Hence, DNA of *A. paragallinarum* were isolated from infectious coryza killed vaccine, produced by ventri biologicals, Pune which contains page reference strains 0083 (A-1), and Modesto (C-2). The primers used in the present study were described by previous workers [6] and it was successfully used by many others worldwide [12,15-17]. A 30 cycle PCR reaction with annealing temperature of 58°C for 1 min was found to be optimum for amplification of 500 bp products.

The samples collected from fresh outbreaks within two to three days of onset of clinical signs where the birds were at acute stage of disease and before the commencement of antibiotic treatment (samples from Vijayawada and Tenali) showed more percentage of positive results than samples collected from the flocks which were ailing from the disease for few weeks and those with antibiotic treatment (ciprofloxacin/enrofloxacin). Samples from the flock from Dwarapudi, which were at the convalescent stage of disease due to effective antibiotic treatment showed all negative for PCR test. Samples from the acute stage of the disease for accurate diagnosis of infectious coryza were recommended by previous workers [3]. Antibiotic treatment significantly reduced the capacity of both conventional cultural examination and PCR test to detect *A. paragallinarum* [4]. The better performance of PCR against cultural examination for field samples probably reflects the difficulties in obtaining samples of good enough quality to ensure the growth of fragile *A. paragallinarum in-vitro* [4]. The false negative results and expense of test can be significantly reduced, if the PCR test is being applied as a flock test as recommended by previous workers [18] by pooling of samples from two to three birds instead of examining samples from individual birds separately.

Two flocks of Aseel chicken, one was backyard flock and the other was exclusively reared for cock fighting found to have suffering from infectious coryza. The flock from BN Kandriga, which was a backyard flock, was experiencing relapse of disease outbreaks at frequent intervals for past 6 months with high morbidity and mortality in freshly hatched out chicks. This flock was being treated for coccidiosis and was housed in a congested and unhygienic shed along with goose, ducks and guinea fowls which were showing no signs of disease. The ducks were found refractory to experimental infection [19]. A previous survey conducted [20] on infectious coryza in native chicken in Indonesia reported eight to 30% positive rate of antibody against *A. paragallinarum* and stressed the need of vaccination against infectious coryza in native chickens of Indonesia. *A. paragallinarum* was isolated from a game cock from Metro Manila district of Philippines [21]. Four isolates of *A. paragallinarum* were obtained from native Kampung chickens of Indonesia and observed that infectious coryza can be present in less intensive production systems [15].

The potential importance of infectious coryza in less intensive system was supported by the fact that infectious coryza killed more chicken than any other disease, including Newcastle disease, in Thai village chickens [22]. Poor housing, parasitism and inadequate nutrition might be the predisposing factors of infectious coryza [7]. The disease in Aseel chicken can pose a serious threat to our backyard poultry wealth and also can act as a source of infection to the commercial poultry. Hence, the occurrence of the infectious coryza in Aseel and other indigenous breeds of chicken must be closely monitored and vaccination must be carried out if it is found to be necessary.

## Conclusion

In the present study, three infectious coryza outbreaks were investigated in commercial poultry and two from Aseel chicken. All the outbreaks showed similar symptoms with varied intensity. The birds were showing signs of acute upper respiratory tract infections like coughing, sneezing, nasal discharge, facial edema, edema of wattle and comb and lacrimation and conjunctivitis. PCR was standardized for rapid and accurate diagnosis of infectious coryza and field samples were screened. The disease, infectious coryza which was considered to be a disease of commercial chicken was diagnosed in native Aseel chicken and also in commercial chicken from Andhra Pradesh. The PCR was as an easier and rapid diagnostic tool for infectious coryza and found to be highly sensitive while screening the field samples.

## Authors’ Contributions

BS has planned and designed the study. TMN has conducted the research and analyzed the samples. TMN prepared the manuscript under the guidance of BS. Both authors read and approved the final manuscript
